# Switchable edge-line coupler based on parity time-reversal duality symmetry

**DOI:** 10.1038/s41598-022-20926-w

**Published:** 2022-11-08

**Authors:** Iram Nadeem, Valentina Verri, Enrica Martini, Fabio Morgia, Maurizio Mattivi, Alberto Toccafondi, Stefano Maci

**Affiliations:** 1 Department of Information Engineering and Mathematics, University of Siena, 53100 Siena, Italy; 2 Huawei Technologies, Milan Research Center, Milan, Italy

**Keywords:** Electrical and electronic engineering, Electronics, photonics and device physics

## Abstract

A compact broadband Edge-Line Coupler (ELC) based on Parity Time-reversal Duality (PTD) symmetry has been conceived, designed, constructed and measured. The coupler connects four PTD bifilar edge lines (BELs), recently introduced by the authors. The PTD-BELs are constituted by a parallel plate waveguide whose walls are formed by a junction between Perfect Electric Conductor (PEC) and Perfect Magnetic Conductor (PMC) boundary conditions. Reversing the axis orthogonal to the plates interchanges the position of PEC and PMC. Such a waveguide supports unimodal transverse electromagnetic (TEM) propagation, extremely confined along the top and bottom junction edges; its propagation is protected against backscattering from any discontinuity that preserves the PTD symmetry. The ELC presented here is constituted by a 4-port junction in which each port is intrinsically matched due to the PTD symmetry, strongly coupled with a second port, strongly decoupled with a third port, and weakly coupled with a fourth port. The ELC is designed by using a mushroom metasurface for the PMC portion of the device; the connection is based on a switch circuit which imposes open and short conditions on the two opposite sides of the structure. Switching simultaneously the open and short circuits reroutes the signal in a different port, while maintaining the same level of coupling with the other ports. A static prototype has been built and its measurements have confirmed the matching performance and the good directionality of the coupler in a broadband frequency range between 24 and 30 GHz.

## Introduction

Topological photonics^1^ has recently fascinated scientists in different fields. This new area, formerly developed for electronic devices, has been applied to electromagnetic systems opening new opportunities^2,3^. Topological edge modes (TPEMs) exhibit unidirectional propagation along an edge, without any reflections from physical imperfections^1^. The TPEMs unidirectionality requires nonreciprocal elements^4–6^, which make the system complex. However, recent researches have shown that a robust propagation can also be achieved in reciprocal structures when they exhibit a parity (P), time-reversal (T), duality (D) symmetry. It was first shown in^7^ that an N-port network invariant under the PTD operator is protected against back-scattering from the class of discontinuities that respect the same PTD symmetry property. Interestingly, PTD symmetry can be achieved also without bulk magnetic materials, since duality can be played on boundary conditions by using metasurfaces. These latter, in fact, can be conveniently used to manipulate electromagnetic fields^8–11^. In few words, a passive and lossless reciprocal waveguide (WG) is PTD-symmetric when applying a symmetry operator to its geometry renders the boundary conditions dual in the Babinet’s sense. If this is obtained, the WG is able to support propagation without back-scattering, namely it is intrinsically perfectly matched even in presence of discontinuities occurring along the path. Different symmetry-protected WGs proposed in the literature can be cast in this framework^12–15^. For instance, pairing two semi-infinite parallel plate WGs with swapped Perfect Electric Conductor (PEC) and Perfect Magnetic Conductor (PMC) boundary conditions (BCs) one obtains an edge WG that satisfies PTD-symmetry with respect to the direction normal to the two plates^14,15^. When the separation between the two walls is less than a quarter of wavelength, this structure can support a unique mode strongly confined at the edges between PEC and PMC. For this reason, we denote this guiding structure as bifilar edge line (BEL) (even called Bifilar Edge Waveguide). This PTD-BEL has been numerically and experimentally investigated in^16^.

In this paper, PTD-BELs are used to design a switchable directional coupler suitable for PCB technology realization. Directional couplers are fundamental components for several microwave and millimeter-wave systems, and various solutions have been developed for their implementation^17^. Rectangular waveguide realizations are suitable for high-performance and high-power applications^18^. However, they are bulky, rather expensive and difficult to integrate with other circuit components. On the other hand, branch-line hybrid and coupled-line directional couplers in stripline and microstrip technologies offer the advantages of compactness, planarity, low fabrication cost, and integrability with active devices. In the recent years, substrate-integrated waveguide (SIW) solutions have been developed as a compromise between the previous technologies. These include planar single^19,20^ and multilayer^21^ structures, as well as three dimensional configurations^22^. They normally use slots (multiple apertures or a continuous one, depending on the desired coupling level) in the common wall of two adjacent SIW waveguides. In all the cases above, the couplers are static. However, some applications require the possibility to either reroute the signal towards a different port or change the coupling level of the coupled port. Various approaches have been reported for low-frequency (less than 5GHz) couplers with tunable power division ratio^23–25^ and some other also include direction switching capability^26,27^. In this paper, we present a compact solution for a switchable directional coupler whose design is based on PTD symmetry theory. It consists of 4-port, each one connected to a PTD-BEL. The four BELs are confluent in the center. Exciting one port routes the signal in one port only, without reflection and with a very low coupling to the other ports. The overall structure can be seen as composed by two parallel plates in which the top and bottom plates are divided in four quadrants, which exhibit alternating PEC and PMC BCs. The two plates are superimposed so that the PEC quadrant on the top wall is directly faced with a PMC quadrant on the bottom wall. The central confluence point either on top and on bottom walls possesses undefined BCs that should be forced to be PEC on top wall and PMC on bottom wall (or vice versa) in order to guarantee the PTD symmetry. Swapping PEC and PMC at the central point changes the coupled port, while maintaining excellent matching and directionality. This opens the opportunity of realizing an almost “perfect switch” in which the signal is routed to different ports by switches located at the 4-edge-line central junction. However, synthesizing the PMC with metasurfaces, renders the “perfect rerouting” with a zero-dimension switch impossible to be realized. Despite this obvious impairment, enlarging the central zone to a minimum of 4 periodic metasurface cells provides a good large bandwidth switchable coupler.

A static prototype has been realized, in which the PMC portions of the device are implemented via a high-impedance mushroom metasurface^28^. The structure is then successfully measured, showing scattering parameters in excellent agreement with the PTD theory. In the realization, the structure is excited by a grounded-coplanar waveguide (G-CPW) coupled to a substrate integrated waveguide (SIW) to benefit from the printed circuit board (PCB) technology. Compared with other switchable couplers presented in the literature, like^26,27^, the proposed solution is shown to be able to operate at higher frequencies (up to the Ka-band) and over a larger bandwidth, with good performances in terms of matching, isolation and efficiency.

The presentation of the paper is organized as follows. Section “The presentation of the paper is organized” illustrates the basic concept, making reference to an ideal structure. Section “Design based on mushroom metasurfaces” shows a design with mushroom metasurfaces. Section “Prototyping and measurements” describes the constructed prototype and presents the measurements of the scattering parameters in the range 24-30 GHz. Conclusions are drawn in Section “Concluding remarks”.

## Ideal PEC-PMC Case

**Figure 1:**
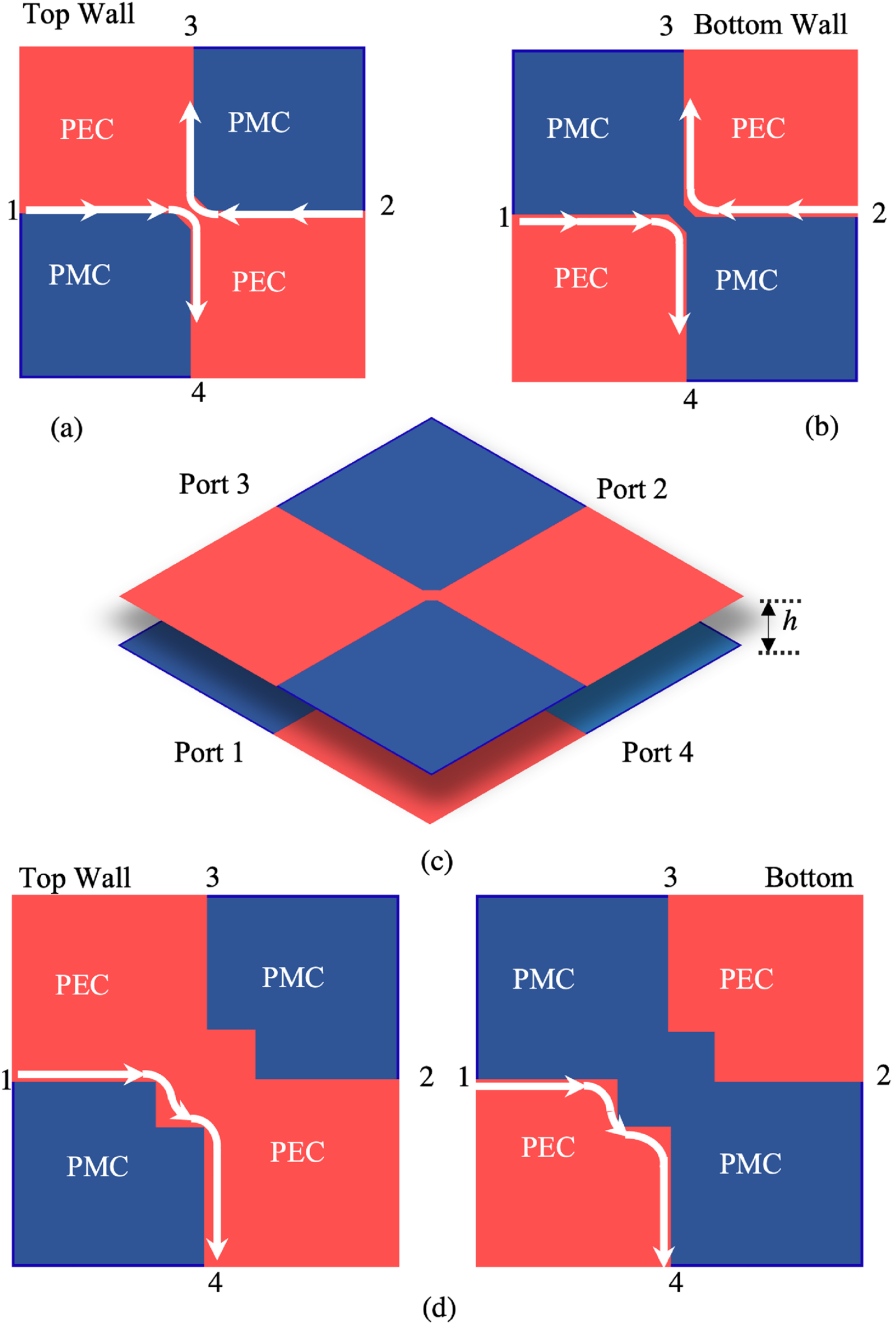
Ideal 4-ports coupler composed by a parallel plate with four alternating PEC/PMC squares on top (**a**) and dual wall on the bottom (**b**). A small bridge at the central connection maintains the PTD-symmetry of the device with respect to the orthogonal axis.(**c**) Three dimensional view of the coupler. (**d**) Alternative geometry with a larger central part.

Consider a geometry consisting of a parallel plate structure with distance *h* between the two plates. Each plate has four quadrants alternating PEC and PMC BCs (Fig. 1). In pairing the top and bottom walls, the PEC and PMC quadrants are placed one on top of the other (see Fig. 1a,b). This structure is PTD symmetric with respect to the axis *z* orthogonal to the plates (“parity axis”), since changing *z* in *-z* makes the BCs dual. This arrangement can also be seen as a 4-port junction constituted by the confluence of four PTD-BELs, a kind of edge WG which has been theoretically studied in^14^ and experimentally verified in^29^. This transmission line is robust to back-scattering for any PTD symmetric discontinuity, like the one at the central point of the junctions. Therefore, according to the PTD theory, the $S_{ii}, i=1..4$ parameters at the input ports are rigorously zero, namely, the 4-port junction is perfectly matched at the ports.

However, it is important to note an aspect which has an impact on both modelling and practical implementation: the BCs at the connection point at the confluence of the four PTD-BELs is undefined. If one does not properly attribute the BCs at this point, this implies a violation of the PTD symmetry. To guarantee a PTD symmetry, it is necessary to attribute opposite BCs on the top and bottom walls at the connection point, for instance PEC on the top wall and PMC on the bottom wall (Fig. 1). In this way, the complementary behavior when swapping *z* in $-z$ is ensured, and therefore the matching property at the 4 ports is guaranteed. In practice, the attribution of the BC to the central point of the junction is done on the top wall by connecting the PEC quadrants with a small PEC bridge (see Fig. 1a), and on the bottom wall by separating PEC quadrants by a PMC gap (see Fig. 1b). The attribution of complementary BCs at the central point not only renders the structure PTD-symmetric, but it also defines edge-line paths between Ports 1 and 4 and between Ports 2 and 3, as shown by the arrows in Fig. 1a,b. These arrows represent the flow of electric currents, essentially concentrated on the metallic part close to the edge. The magnetic currents follow the same edge driven path on the PMC part. It is also worth noting that this behavior is independent of the size of the central bridge. Therefore, for practical implementation, one can also increase the size of the central part, as represented in Fig. 1d. The current will still flow along the edges without any reflection. As we will see next, this arrangement allows to better control the coupling factor to port 2 in practical realization.

**Figure 2:**
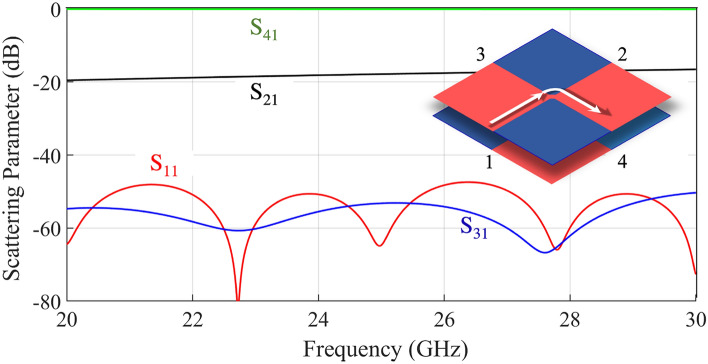
Simulated scattering parameters of the ideal 4-ports junction shown in Fig. 1c.

Figure 2 shows the scattering parameters $S_{ij}$ obtained by a numerical full-wave analysis, performed through Simulia CST for the case in Fig. 1c. Ideal BCs are assigned to the two walls, which are separated by a distance $h = 1$ mm. Port 1 is fed by exciting the modal field distribution at the corresponding section. The S-parameters shows that Port 1 is almost perfectly matched and nearly all the input power is delivered to Port 4. Isolation of Port 3 is very high, only limited by the numerical accuracy, and the coupling to Port 2 is around 20 dB in all the considered bandwidth. The directivity of this coupler (power at port 2 over the power at port 3) is therefore more than 35 dB in all the bandwidth.

**Figure 3:**
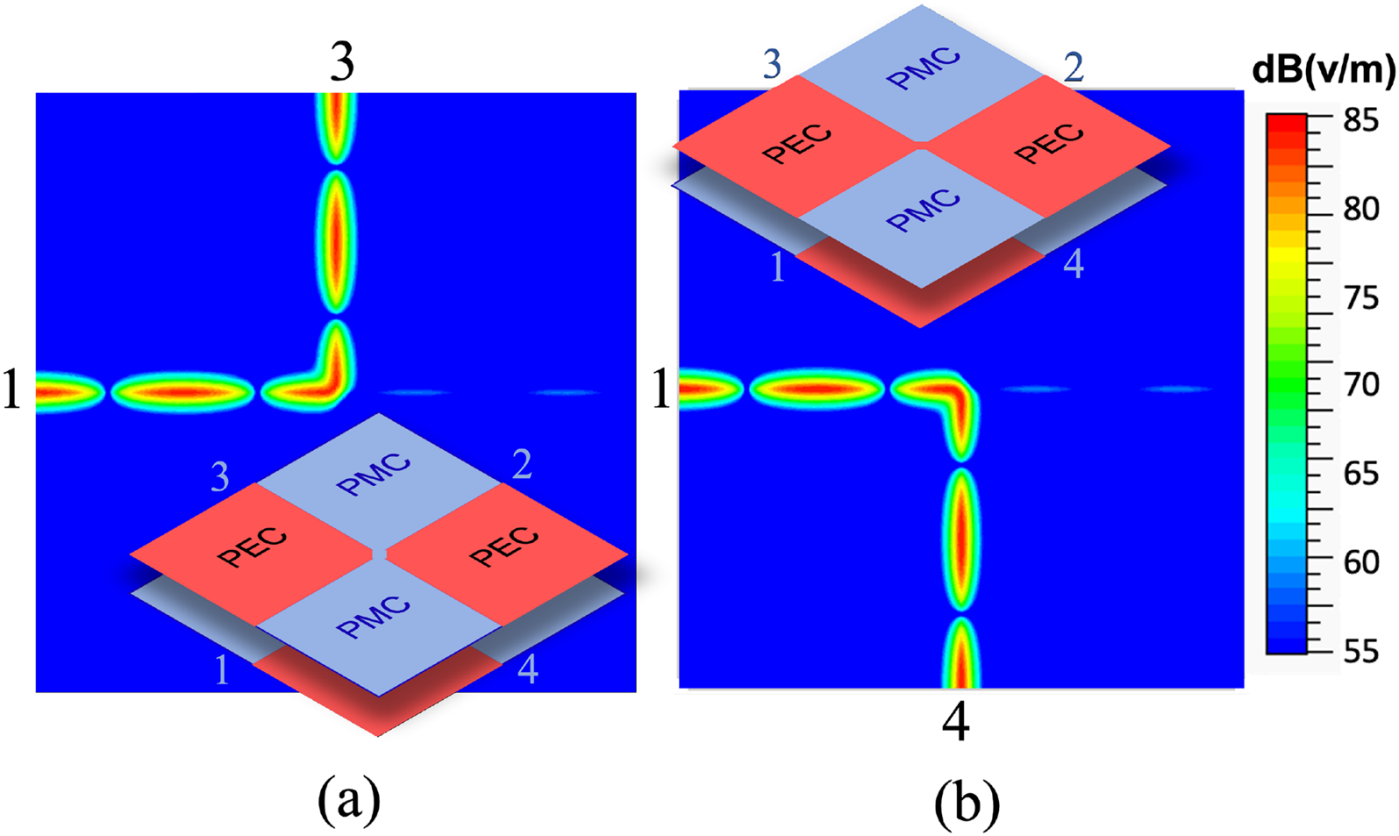
Amplitude of the electric field in the ideal 4-Ports junction for dual BCs on the central gap, as shown in the insets, simulated with Ansys HFSS 2020^30^.

The remarkable thing is that the behavior can be completely changed by only acting on the central connection point. In fact, if the PEC and PMC BCs of the central bridge are swapped, Port 1 will be connected to Port 3, and Port 2 to Port 4. Figure 3 illustrates the amplitude of the electric field for the two different conditions of the central connections: in the first case, power supplied to Port 1 is delivered to Port 3 and weakly coupled to Port 2, with a high isolation of Port 4. In the second case, power supplied to Port 1 is delivered to Port 4 and coupled to Port 2, with a high isolation of Port 3. In both these cases, the return loss is excellent. This means that it is possible to re-route the signal by changing the nature of a very small region at the junction central point. This can be done in practice by using simple switching elements like PIN diodes.

## Design based on mushroom metasurfaces

**Figure 4:**
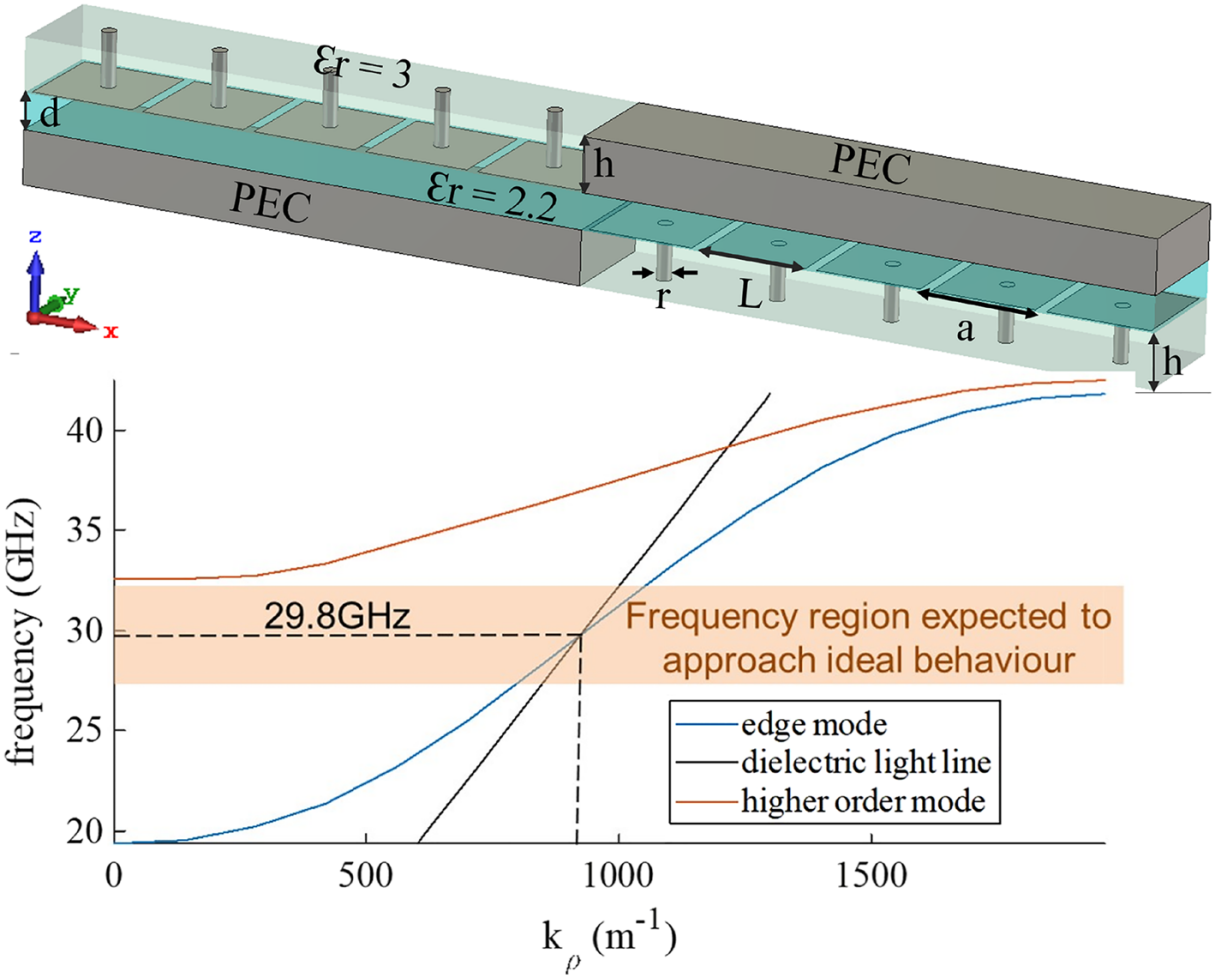
Geometry for the unit cell of the PTD-symmetric BEL and relevant dispersion diagram. The crossing point between the edge mode dispersion curve and the light line in the filling dielectric corresponds to the central operating frequency, where the mushroom metasurface behaves like a PMC.

The practical implementation of the ideal 4-ports junction in Fig. 1 is obtained by using a mushroom type metasurface to emulate PMC. The unit cell of this metasurface consists of a square patch printed on a grounded dielectric substrate Rogers RO3003, characterized by relative permittivity equal to 3 and slab thickness $h=0.762$ mm. It is connected to the ground plane through a central via, as shown in the inset of Fig. 4. A parametric study has been carried out in order to optimize the mushroom unit cell. Finally, the following geometrical parameters have been selected: size of the unit cell $a=1.6$ mm, side of the square patch $L=1.45$ mm, via radius $r=0.1$ mm. The space between the PEC wall and the top of the mushroom is filled with a dielectric with relative permittivity $\epsilon _r=2.2$ and has a thickness $d=0.508$ mm. The resulting structure has been analyzed with Simulia CST.

The dispersion diagram reported in Fig. 4 shows that the light line crosses the dispersion curve around 30 GHz; this is the frequency where the mushroom metasurface better emulates the PMC. Around this point, the BEL mode is quasi-TEM. In fact, for the ideal PEC-PMC BCs, the supported mode is TEM^14,15^, but the practical implementation of the PMC through mushrooms introduces a dispersion effect, so that the mode will be quasi-TEM. The unimodal bandwidth ranges from 19.36 GHz (the cut-off frequency of the edge mode) to 32.57 GHz (the cut-off frequency of the first higher order mode), corresponding to a relative bandwidth of approximately $50\ \%$. However, the relative bandwidth in which the structure is expected to approach the ideal behaviour, represented by the shaded area in Fig. 4), is around $20\,\%$.

The PTD coupler concept is verified by emulating the PMC with mushrooms using the BELs defined above. Due to the finite dimension of the mushroom unit cell, we cannot create only a small-bridge at the confluence point, but we must have a finite region consisting of a certain number of unit cells. Therefore, the structure in Fig. 1d describes the practical implementation of the ideal switchable coupler better than the one in Fig. 1c.

**Figure 5:**
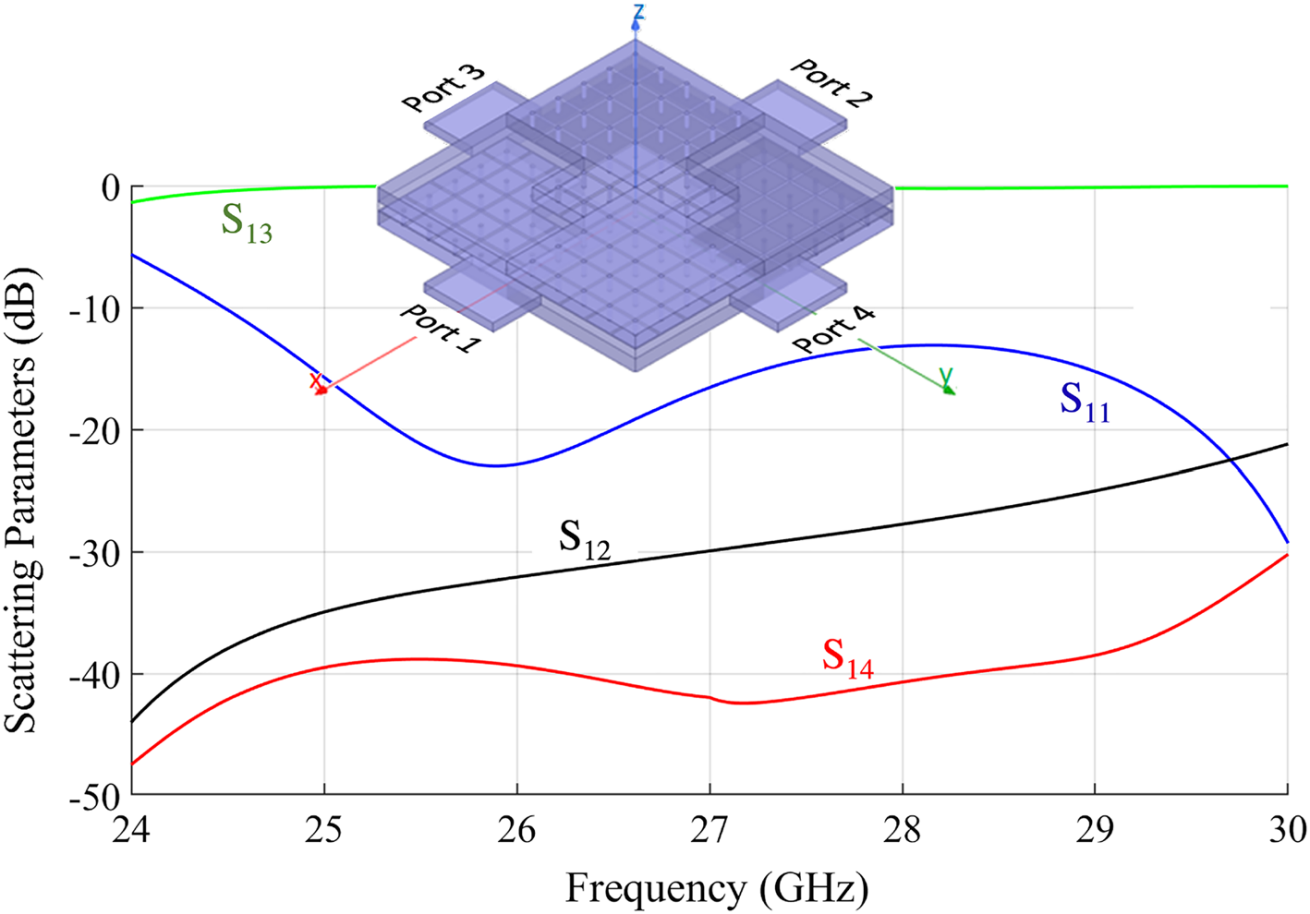
Simulated scattering parameters for the ELC coupler implemented through mushroom metasurfaces with 4x4 unit cells in the central part.

Two designs have been analyzed. In the first design, a 4x4 unit cell region is defined at the center, with mushroom distribution on the top wall and metal on the bottom. The structure is illustrated in the inset of Fig. 5. The arrangement is such that the directionality is from Port 1 to Port 3 and from Port 2 to Port 4. The simulated scattering parameters for this structure are shown in Fig. 5. It can be seen that in the considered frequency range almost all the power fed at Port 1 is delivered to Port 3 with a good return loss. The coupling factor to Port 2 ranges from 35 dB up to 20 dB in the bandwitdh. Isolation of port 4 is almost constant around 40 dB up to 29 GHz.

**Figure 6:**
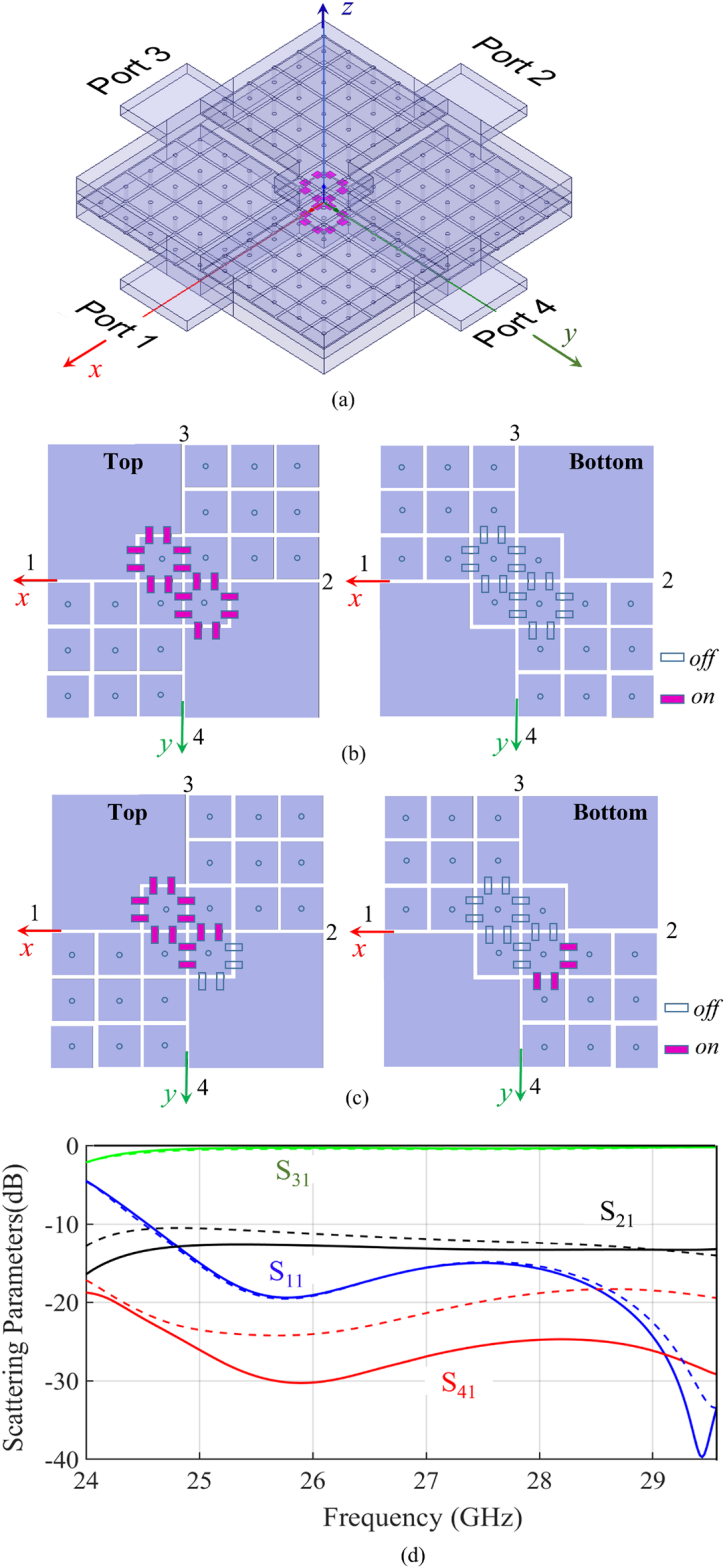
(**a**) Switchable coupler realized by using 2x2 mushroom unit cells in the central part both on the top and the bottom walls; (**b**), (**c**) two different configurations of the pin-diodes (**d**) Simulated Scattering parameters obtained by using HFSS^30^ for the two configurations: (**b**) solid lines (**c**) dotted lines.The full wave analysis includes the waveguide ports, as shown in (**a**).

**Figure 7:**
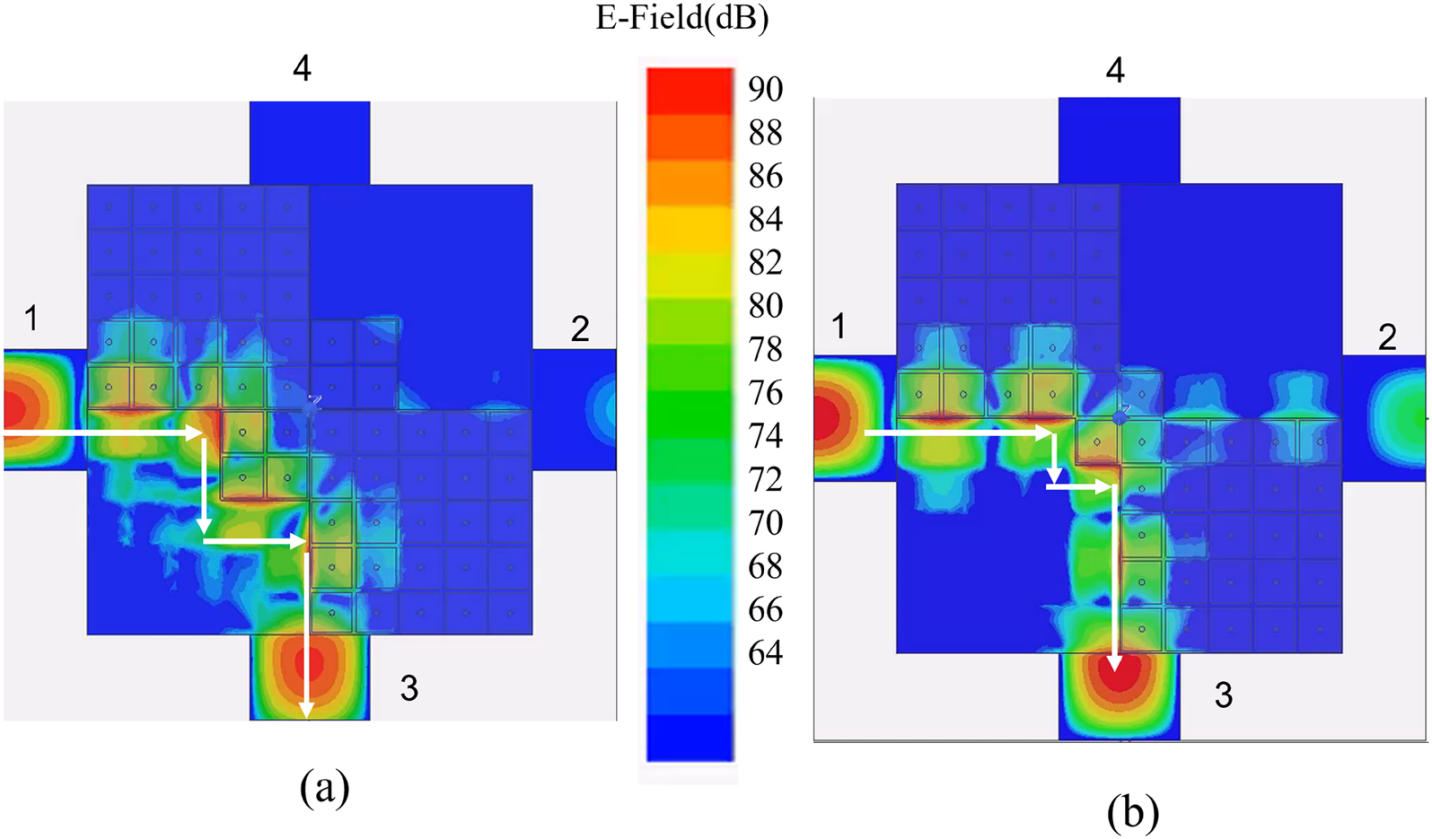
Amplitude of the electric field at 29.8 GHz simulated with Ansys HFSS 2020^30^, (**a**): 4x4 unit celles in the central region (design of Fig. 5); (**b**): 2X2 cells in the central region (design of Fig. 6b).

A second design is shown in Fig. 6a. In order to realize a reconfigurable device for signal routing it is necessary to switch the behavior of the central region from PEC to PMC. This is implemented by setting 2x2 mushroom unit cells in the central region on both the top and the bottom walls, and by introducing switchable connections (PIN diodes) among adjacent patches (see Fig. 6b,c). When the diodes are on, they short-circuit the patches, thus emulating the PEC behavior. When the diodes are off, they act as small capacitance, thus, leaving substantially unaffected the PMC-type mushroom behavior. In the full-wave analysis, the diodes are first emulated with ideal short or open circuits; in practice, they can be biased by using channels inside the pins of the mushroom^31^. Figures 6b,c show two different configurations for the states of the diodes and Fig. 6c presents the relevant scattering parameters as simulated by Ansys HFSS v2020^30^. Rectangular waveguides (RWGs) have been used to feed the ports, and have been included in the numerical simulation; the thickness of the RWGs has been fixed equal to the distance between the mushroom surface and the PEC walls (i.e., 0.508 mm), while their width is set to 4.3 mm, which is the value that provides the best matching to the BEL. As expected, in both the configurations the power supplied to Port 1 is delivered to Port 3, and the return loss is quite good. In the case of Fig. 6b, isolation of Port 4 is better than 25 dB in the frequency range $25-29.5$ GHz and the coupling level of Port 2 is constantly around 12 dB in the same bandwidth. As a consequence, the directivity of this coupler is better than 13 dB, quite stable in the mentioned bandwidth. The configuration in Fig. 6c exhibits a similar behaviour, but with a slightly increased coupling to port 2 and a lower isolation. This shows how the switches can not only reroute the signal with good matching, but also control the amount of power transmitted to the ports.

Comparison with the results of Fig. 5 shows essentially a different coupling level of Port 2, due to the different number of cells used in the central region. Fig. 7 shows snapshots of the electric field distribution at 29.8 GHz for the structure in Fig.5 and for the one in Fig. 6a. We remark that the difference between the two structures in Fig. 7 is not only that the first one (inset of Fig. 5) has a larger central area (4x4 cells vs 2x2 cells) but also that in the second one (Fig. 6) the PEC in the central area is recreated by connecting diodes across the mushroom elements. In both cases, the signal is delivered from Port 1 to Port 3 with a small coupling factor to Port 2. The main difference in the scattering parameters is the value of the coupling factor, which is controlled by the dimension of the central part (the larger the central, the smaller the coupling factor toward port 2). It is clear by symmetry that switching the state of the diodes re-routes the signal from Port 1 to Port 4 with the same coupling factor to Port 2.

**Figure 8:**
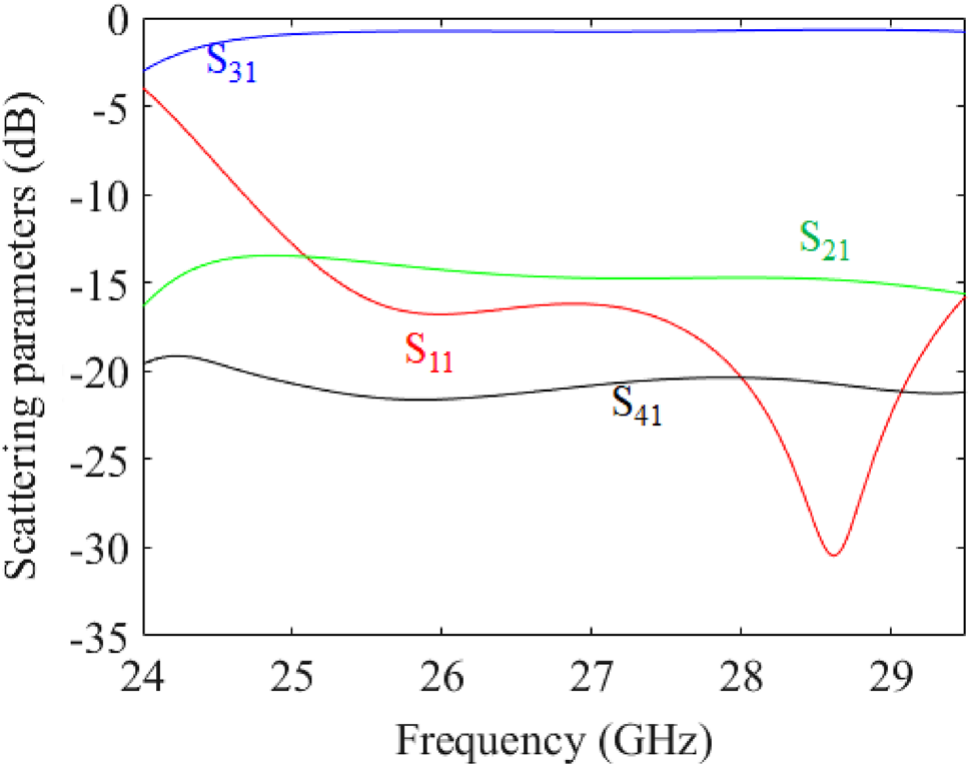
Simulated scattering parameters for the structure in Fig. 6b with a realistic model for the PIN-diodes, accounting for the main parasitic effects.

Finally, a more realistic model has been considered for the PIN diodes, to assess the impact of parasitic effects on the device performances. These effects in general depend on PIN diode type and package. Here, we have considered a commercially available surface mount PIN diode (Macom MA4FCP200) able to operate as a Single Pole Single Through switch up to 40 GHz. In accordance with the information provided in the data sheet^32^, we have modelled any forward biased PIN diodes as a $2.8\,$Ohm resistance and any reverse biased PIN diode as a $20\,$fF capacitance. The results of the relevant full wave simulations for the configuration in Fig. 6b are reported in Fig. 8. Simulation results show that parasitic effect cause a small deterioration of the performances, especially in terms of reduced isolation and increased losses but, overall, they do not significantly affect the behavior of the proposed device. This confirm that, with the actual state-of-the-art PIN-diodes, the proposed configuration is feasible up to the Ka-band. It is apparent that non ideality of the switches has a smaller impact at lower frequencies.

## Prototyping and measurements

**Figure 9:**
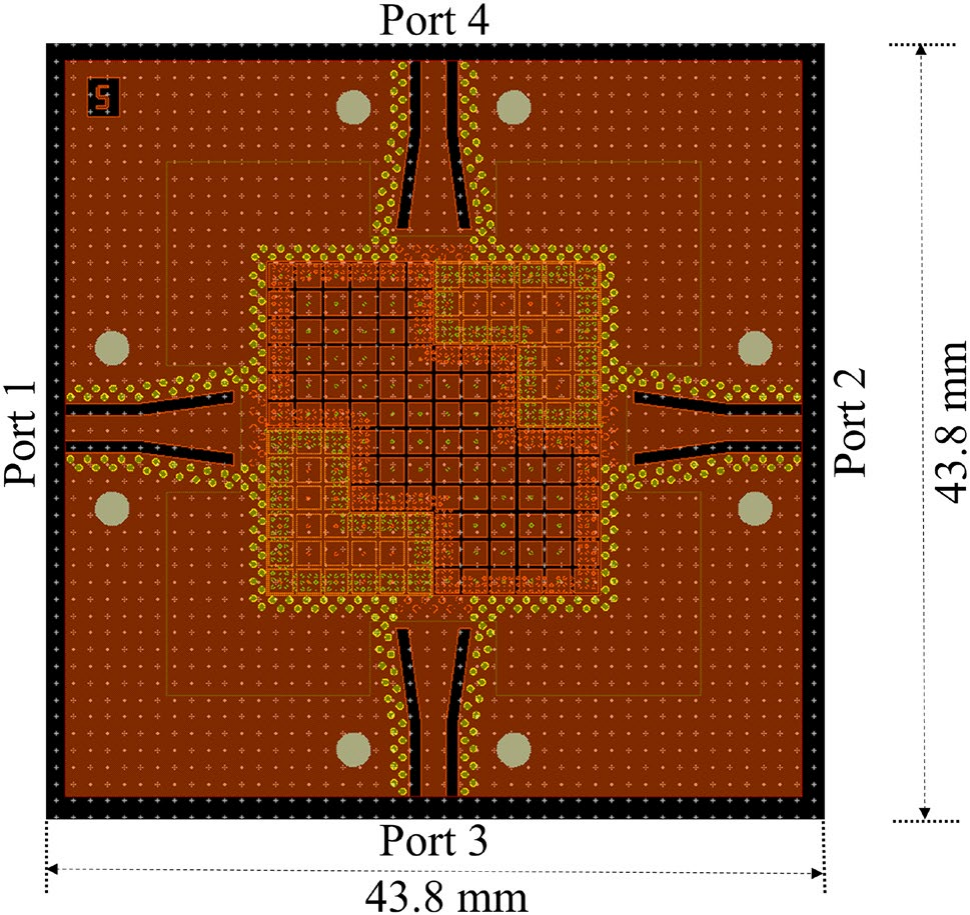
Layout of 4-ports PTD-Symmetric ELC by using grounded-coplanar waveguide to a substrate-integrated waveguide transition.

A prototype of the four ports PTD-Symmetric ELC shown in Fig. 5 has been realized in PCB technology. Three layers of dielectric materials are glued together by means of two layers of Astra MT77 Prepreg 1035LD of thickness 0.126 mm each. The first dielectric layer is a Rogers RO3003 of thickness 0.762 mm, with a top metallization made by copper of 0.06 mm and a bottom one of 0.05 mm. The third layer is made of the same substrate as the first one. In between these two RO3003 slab layers (used for the realization of the mushroom metasurface) there is a layer of Rogers RT5870, which has relative permittivity $\epsilon _r=2.33$ and loss tangent equal to 0.0012. The central part consists of a square region with 3x3 mushroom MTS unit cells on top and a PEC block in the bottom as shown in the layout of Fig. 9.

The examples discussed in the previous sections are with RWG feeding. In order to realize the structure in PCB technology, with an easy coupling to an SMA connector, we have considered a grounded coplanar input port, complemented by a transition from grounded-coplanar waveguide (G-CPW) to substrate-integrated waveguide (SIW)^33–35^. The strip of the G-CPW is printed at the same level of the mushroom surfaces patches. Taking into account the additional thickness due to the presence of the prepreg, the thickness of the RT5870 was taken equal to 0.254 mm in order to achieve performance similar to the ones obtained without prepreg and a thickness of $d=0.508$ mm.

**Figure 10:**
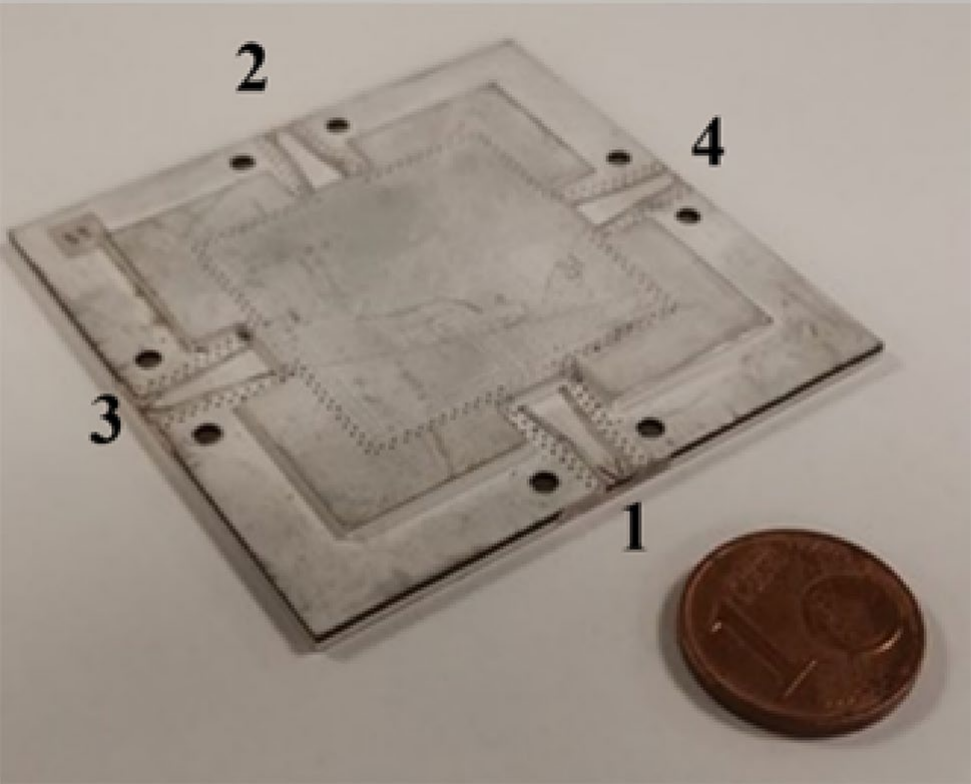
Picture of the realized prototype.

**Figure 11:**
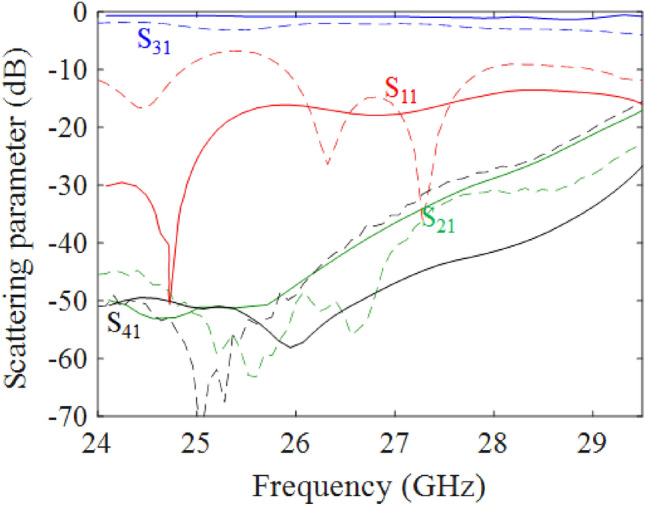
Simulated (continuous lines) and measured (dashed lines) scattering parameters of the PTD-Symmetric ELC using grounded-coplanar waveguide substrate-integrated waveguide transition for excitation at Port 1.

The realized prototype is shown in Fig. 10). The entire structure with all the feeding transitions has been simulated by a full-wave solver (Ansys HFSS 2020^30^) and the results for the scattering parameters when power is supplied to port 1 are shown in Fig. 11 with continuous lines. The corresponding measured scattering parameters are reported in the same figure with dotted lines. The trend of the measured curves is similar to the one obtained by numerical simulation, with less satisfactory return loss (around $-10$ dB) and slightly higher insertion loss, probably due to imperfection in the fabrication.

**Figure 12:**
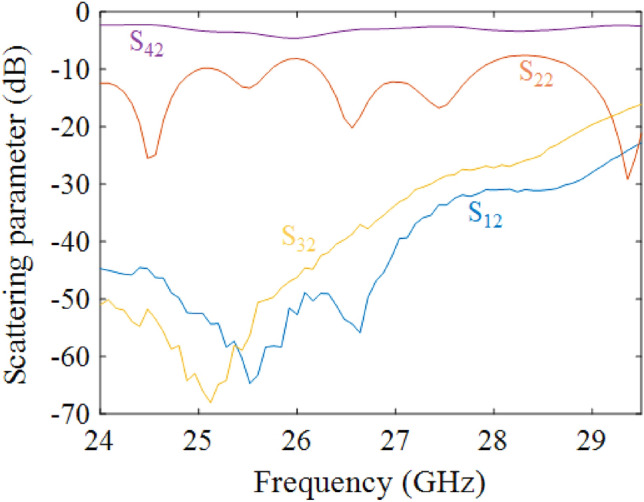
Measured scattering parameters of the PTD-Symmetric ELC using grounded-coplanar waveguide substrate-integrated waveguide transition for excitation at Port 2.

Measured scattering parameters for excitation at Port 2 are reported in Fig. 12. Since, the overall structure is PTD-invariant by swapping the vertical axis, Port 1 is connected only with Port 3, and Port 2 only with Port 4. The measured scattering parameters clearly show this symmetry.

## Concluding remarks

A PTD-invariant edge-line switchable coupler has been presented. In the ideal case of PEC and PMC boundary conditions, a couple of ideal synchronized switches at the confluence among the bifilar edge-lines can re-route the signal from one port to another with excellent matching isolation to one port and very small spill-over to another port. This realizes a very good switch over an extremely large bandwidth. Of course, the need to approximate PMC with practical metasurfaces (like mushroom) imposes a certain dimensions to the central switching region. The minimum dimension that one can realize in practice consists of 2x2 mushroom cells. It is seen in this case that a combination of switches implies strong coupling to one port, high isolation to the opposite port and a spill-over of power to the front port to a level of $-12$ dB, thus realizing a large bandwidth switchable coupler. Different combinations of switches can also modulate the power coupled to the front port. If the central region is instead constituted by more cells (e.g., 4x4), the structure emulates better the ideal case, and the spill-over of power to the front port reduces drastically, as predicted by the PTD theory. However, rerouting the signal in this case requires a higher number of switches and a control board. Although the implementation of the device did not include switches, the experimental results have not only demonstrated the applicability of the PTD-symmetry concept, but also opened the path to the fabrication of microwave devices competitive in terms of design flexibility and bandwidth performance.

## Data availability

The data that support the findings of this study are available from University of Siena, writing at iram.nadeem@student.unisi.it but restrictions apply to the availability of these data, which were used under license for the current study, and so are not publicly available. Data are however available from the authors upon reasonable request and with permission of Huawei.
